# Chronic rhinosinusitis associated with chronic bronchitis in a five-year follow-up: the Telemark study

**DOI:** 10.1186/s12890-022-02203-8

**Published:** 2022-11-09

**Authors:** Joel Bergqvist, Mogens Bove, Anders Andersson, Linus Schiöler, Geir Klepaker, Regine Abrahamsen, Anne Kristin M. Fell, Johan Hellgren

**Affiliations:** 1 Department of Otorhinolaryngology, Head & Neck Surgery, Sahlgrenska University Hospital, Gothenburg, Sweden; 2 Institute of Clinical Sciences, Sahlgrenska Academy, University of Gothenburg, Gothenburg, Sweden; 3 Department of ENT & Oral Maxillofacial Surgery, NU Hospital Group, Trollhättan, Sweden; 4 COPD Center, Department of Respiratory Medicine and Allergology, Sahlgrenska University Hospital, Gothenburg, Sweden; 5 COPD Center, Department of Internal Medicine and Clinical Nutrition, Institute of Medicine, Sahlgrenska Academy, University of Gothenburg, Gothenburg, Sweden; 6 Occupational and Environmental Medicine, Institute of Medicine, Sahlgrenska Academy, University of Gothenburg, Gothenburg, Sweden; 7 Department of Occupational and Environmental Medicine, Telemark Hospital, Skien, Norway; 8 Department of Community Medicine and Global Health, Institute of Health and Society, University of Oslo, Oslo, Norway

**Keywords:** Rhinitis, Chronic bronchitis, Epidemiology

## Abstract

**Background:**

Chronic rhinosinusitis (CRS) is associated with generalised airway inflammation. Few studies have addressed the relationship between CRS and chronic bronchitis (CB).

**Methods:**

This prospective study over a five-year period aims to investigate the risk of developing CB in subjects reporting CRS at the beginning of the study. A random sample of 7393 adult subjects from Telemark County, Norway, answered a comprehensive respiratory questionnaire in 2013 and then 5 years later in 2018. Subjects reporting CB in 2013 were excluded from the analyses. New cases of CB in 2018 were analysed in relation to having CRS in 2013 or not.

**Results:**

The prevalence of new-onset CB in 2018 in the group that reported CRS in 2013 was 11.8%. There was a significant increase in the odds of having CB in 2018 in subjects who reported CRS in 2013 (OR 3.8, 95% CI 2.65–5.40), adjusted for age, sex, BMI, smoking and asthma.

**Conclusion:**

In this large population sample, CRS was associated with increased odds of developing CB during a five-year follow-up. Physicians should be aware of chronic bronchitis in patients with CRS.

## Background

Chronic rhinosinusitis (CRS) is a prevalent, multifactorial upper airway disease affecting up to 12% of the adult population worldwide [1]. CRS is characterised by mucosal inflammation in the nose and paranasal sinuses, causing symptoms of nasal obstruction and discharge, loss of smell, facial pain, malaise resulting in sleep impairment and poor quality of life [2]. CRS has been strongly associated with asthma in both epidemiological and clinical studies [3, 4]. CRS has also been linked to chronic obstructive pulmonary disease (COPD), but there are still only a few studies [3, 5, 6].

Chronic bronchitis (CB) is a long-term inflammatory disease in the lower airways which involves a productive cough, impaired quality of life [7], an increased decline in lung function [8, 9], a higher risk of cardiovascular disease [10] and increased mortality [10]. The worldwide prevalence of CB is estimated to be 6.4% [11]. In the Nordic countries, the prevalence of CB is reported to be 4.6% in Norway and 7.2% in Sweden respectively [12, 13]. Since both CRS and CB are characterised by inflammation in the airway mucosa and have similar co-morbidities and risk factors such as smoking [12, 14] and occupational exposure [15–17], it is of great interest to further investigate the potential relationship between these diseases.

Few studies have previously investigated the association between upper airway inflammation and CB [18, 19]. In a large, population-based study from Sweden, Montnémery et al. showed, using a cross-sectional design, that nasal symptoms were more common in subjects with CB, but they did not use the European Position paper on Rhinosinusitis and Nasal polyps (EPOS) definition of CRS, since this definition was not established at the time. Further, in a small clinical trial, Håkansson et al. subsequently reported that CRS with nasal polyps was associated with CB [18].

To our knowledge, the present study is the first prospective study to assess CRS based on the EPOS definition and the risk of developing CB in a large, randomised population. Additionally, we analysed sex, age, body mass index (BMI), smoking and asthma as possible confounders for developing CB.

## Methods

### Study population

The Telemark Study is a prospective longitudinal study of a random sample from the general population of Telemark County, Norway, designed to evaluate airway diseases in relation to various risk factors such as occupational exposure, lifestyle factors and smoking status. The study has previously been described in detail [20]. Briefly, in 2013, a random sample of 50,000 subjects living in Telemark County in southeastern Norway, aged 16–50, was drawn from the Norwegian population register and a questionnaire was sent out by mail. The response rate after two reminders was 33% (*n* = 16,099). A total of 7952 subjects answered the questionnaire both in 2013 and 5 years later in 2018. The vast majority of the subjects were Caucasians. No clinical examination by a physician were performed.

### Study design

**Fig. 1:**
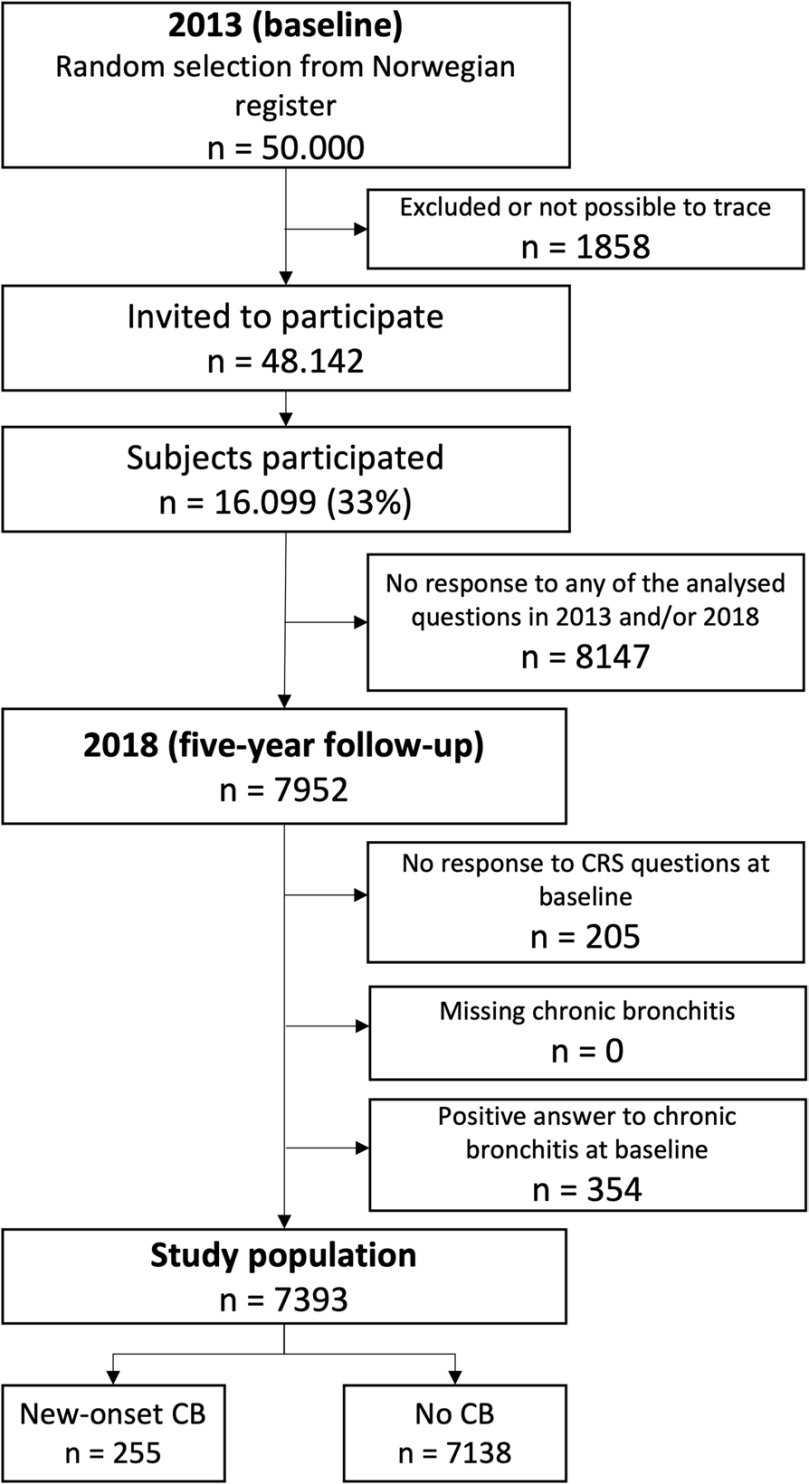
Study flow chart

The present study evaluates the risk of developing CB during a five-year observation period between 2013 and 2018 in relation to having CRS in 2013. All subjects reporting CB in 2013 and subjects who did not answer the questions on CRS in 2013 or 2018 were excluded. A total of 7393 subjects were included in this study. CRS in relation to CB adjusted for potential confounders, such as age, sex, BMI, smoking and asthma, was calculated. All variables and their definitions included in this study are shown in Table 1. A schematic description of the study design is shown in Fig. 1.

**Table 1:** Baseline demographic data and clinical characteristics in 2013

	No CRS (***n*** = 6901)	CRS (***n*** = 492)	Total (***n*** = 7393)	Missing (n)
Baseline data				
Age (years, median (IQR))	41 (17–50)	42 (17–50)	41 (17–50)	–
BMI (kg/m^2,^ median (IQR)))	24.9 (14.1–61.8)	25.6 (17.9–67.6)	24.9 (14.1–67.6)	1307
Sex, n (%)				
Male	2835 (41.1)	214 (43.5)	3049 (41.2)	–
Female	4065 (58.9)	278 (56.5)	4344 (58.8)	–
Smoking, n (%)				21
Current	1354 (19.7)	119 (24.2)	1473 (20.0)	
Past	1546 (22.5)	127 (25.9)	1673 (22.7)	
Never	3981 (57.8)	245 (49.9)	4226 (57.3)	
Asthma, n (%)				109
Yes	586 (8.6)	101 (21.3)	687 (9.4)	
No	6223 (91.4)	374 (78.7)	6597 (90.6)	

*BMI* body mass index

### Outcomes

*Chronic bronchitis* was defined as the positive answer to all three questions, 1, “Do you usually cough up phlegm or have mucus in the lungs that is hard to get up? 2. Do you cough up or bring up phlegm in this way nearly every day for at least 3 months each year? 3. Have you had periods with similar symptoms for at least two consecutive years? [21, 22].

**Fig. 2:**
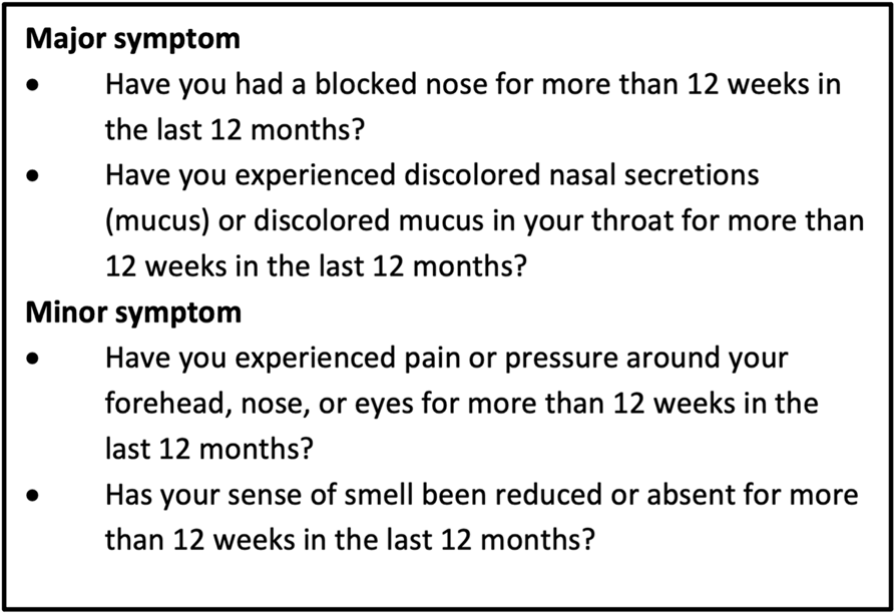
Questions defining chronic rhinosinusitis

EPOS guideline is a widely accepted consensus document. The EPOS guidelines have a validated definition of CRS for epidemiological studies used in this study [2]. *CRS* was defined as the presence of two or more CRS cardinal symptoms for ≥12 weeks, of which one must be either nasal blockage/obstruction/congestion or nasal discharge (anterior/posterior nasal drip), called “major symptom”, and/or additional symptoms, such as facial pain/pressure and/or a reduction in or the loss of smell, called “minor symptom”. All subjects giving a positive answer to at least two major symptoms and/or one major and one minor symptom were defined as having CRS. Clinical studies of *chronic rhinosinusitis (CRS)* recommend the performance of a clinical examination by a physician, including an endoscopic examination of the nose and a CT scan of the nose and sinuses. The specific questions defining CRS is seen in Fig. 2.

In a sub-analysis, we explored whether there was a relationship between the number of CRS cardinal symptoms reported and the likelihood of developing CB during the five-year observation period. Subjects who reported CRS cardinal symptoms without complying with the definition of CRS according to the EPOS criteria were categorised as *“CRS-related symptoms”* (for example, one or two minor symptoms). Subjects fulfilling the minimum criteria for CRS were categorised as “*CRS*” and, finally, subjects who reported more cardinal symptoms than the minimum criteria for CRS were categorised as *“CRS+”* (for example, two major and two minor symptoms).

*Asthma* was defined as a positive answer to the question “Have you ever been diagnosed with asthma by a physician?”

*Smoking status* was defined and categorised as *current smoker, past* and *never smokers,* based on the following questions*: “*Do you smoke daily (applies even if you only smoke a few cigarettes, cigars, or pipe daily)?” and/or “Do you smoke only occasionally (not daily, but weekends, party smoking or the like)” was categorised as *“current smokers”*. “Have you smoked in the past?” was categorised as *“past smoker”*. “*Never smokers”* answered no to all of the questions mentioned above.

The study was approved by the Regional Committee for Medical and Health Research Ethics in Norway (2012/1665/REK Sør-Øst D) and written informed consent was obtained from all the subjects before study inclusion.

### Statistics

Analyses were performed with the SAS statistical software version 9.4 (SAS Institute Inc., Cary, NC, USA). Chi-square tests were used for categorical variables and the Cochran-Armitage trend test was used for unadjusted trend analysis. Multivariable logistic regression models were used to calculate odds ratios and 95% confidence intervals. *P*-values of < 0.05 were considered statistically significant.

## Results

### Study population characteristics

The study population comprises a total of 7393 individuals, all of whom answered the CRS questions in both 2013 and 2018. The prevalence of CRS in the study population was 6.6% in 2013. The median age of the study population in 2013 was 41 years (IQR, 17–50; Table 1). In 2013, both current smoking and asthma were more common in the CRS group (24.2% vs. 19.7%) and (21.3% vs. 8.6%) respectively (Table 1). At follow-up in 2018, 255 subjects reported new-onset CB (Fig.1).

### The risk of developing CB when having CRS

**Fig. 3:**
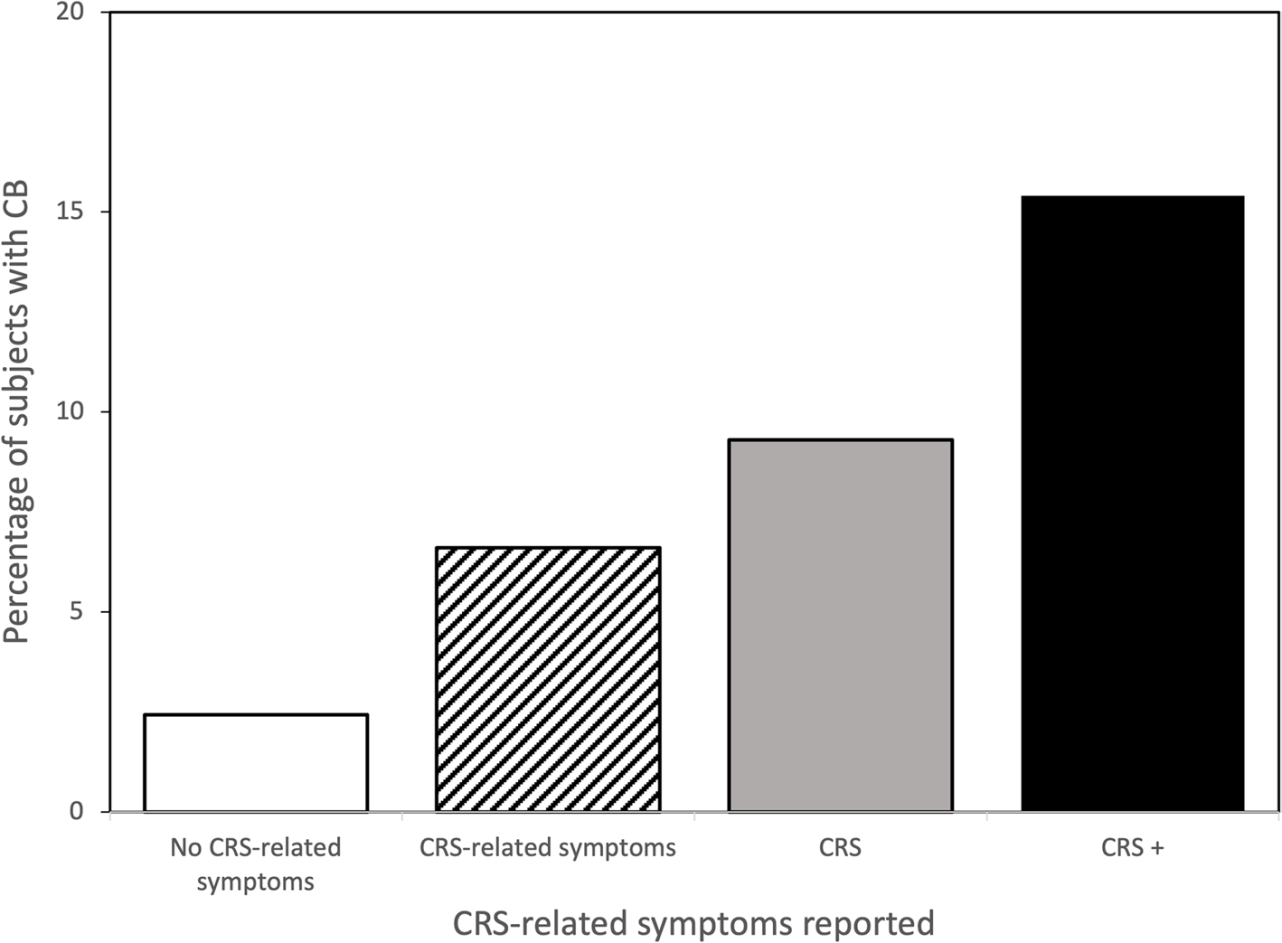
Percentage of subjects with CRS-related symptoms (CRS cardinal symptoms without fulfilling the definition for CRS), CRS and CRS+ (more CRS cardinal symptoms than the minimum criteria for CRS) in relation to chronic bronchitis (CB). Cochran-Armitage trend test *p* < 0.01

**Fig. 4:**
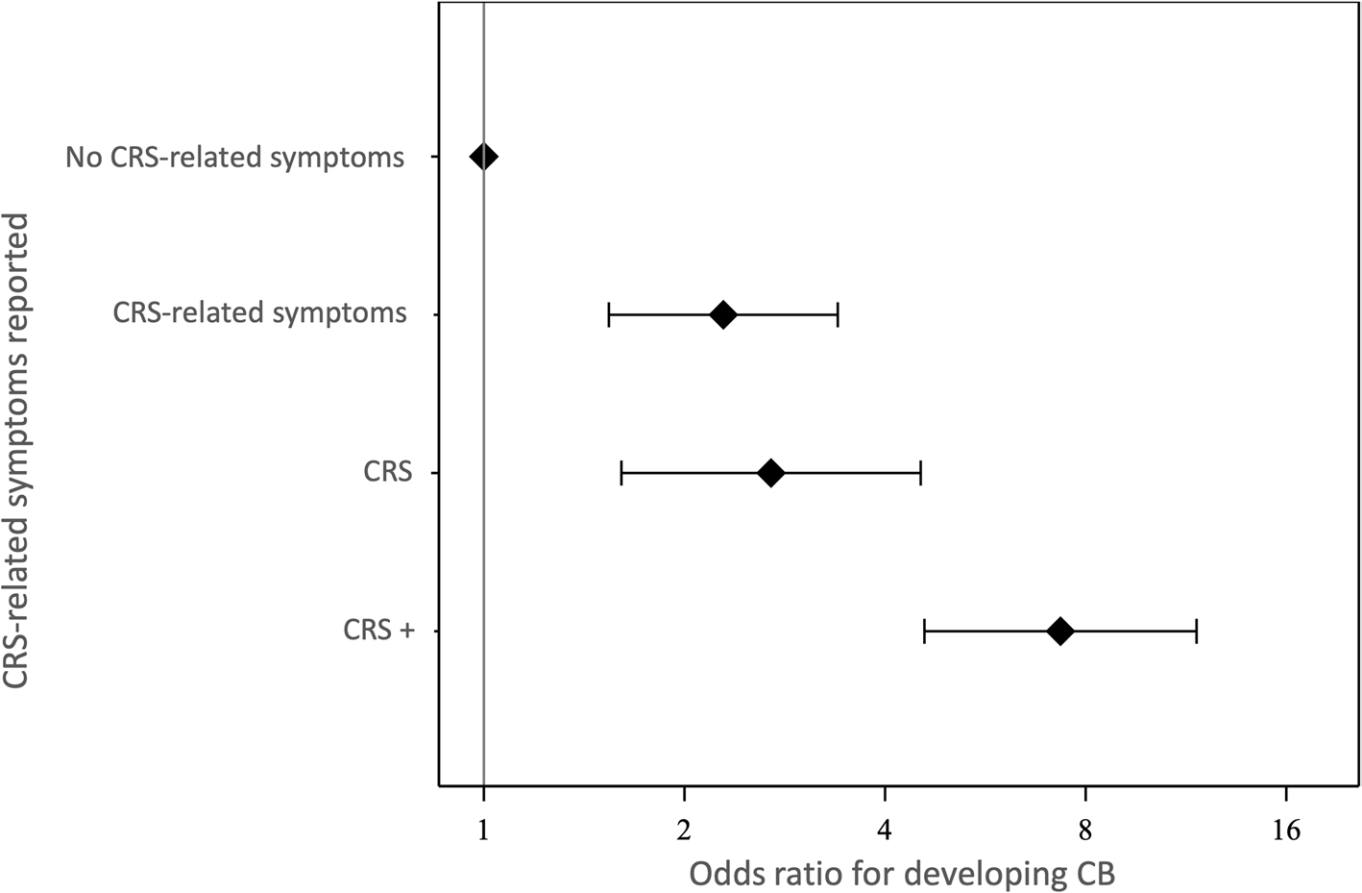
Odds ratio (OR) for developing chronic bronchitis (CB) in relation to CRS-related symptoms (CRS cardinal symptoms without fulfilling the definition for CRS), CRS and CRS+ (more CRS cardinal symptoms than the minimum criteria for CRS) adjusted for age, sex, BMI, smoking and asthma

The prevalence of new-onset CB in 2018 in the group that reported CRS in 2013 was 11.8% (Table 2). There was a significant increase in the odds of having CB in 2018 in subjects who reported CRS in 2013 (OR 3.8, 95% CI 2.65–5.40), adjusted for age, sex, BMI, smoking and asthma compared with subjects who did not report CRS. In the sub-analysis of the relationship between the number of CRS cardinal symptoms and the likelihood of developing CB, the prevalence of CB increased with each category, *CRS-related symptoms, CRS* and *CRS+* (*p* < 0.01; Fig.3). In the logistic regression analysis, the odds were approximately seven times higher in the *CRS+* group compared with not reporting any of the CRS cardinal symptoms (OR 7.33, 95% CI 4.58–11.73; Fig. 4). Age (*p* = 0.02), BMI (*p* = 0.003), smoking (*p* = 0.009) and asthma (*p* < 0.001) but not sex (*p* = 0.6) were regarded as confounders for CB in the regression analysis.

**Table 2:** Chronic rhinosinusitis (CRS) in 2013 and its relationship to chronic bronchitis (CB) in 2018

	No CRS (***n*** = 7138)	CRS (***n*** = 255)	Total (***n*** = 7393)
New-onset CB, n (%)			
Yes	197 (2.8)	58 (11.8)	255 (3.5)
No	6704 (97.2)	434 (88.2)	7138 (96.5)

## Discussion

In this prospective, longitudinal, population-based study, 7393 randomly selected subjects from Telemark, Norway, were followed for 5 years. Having CRS was associated with an increased risk of developing CB during the five-year observation period and, in the multiple regression model adjusted for age, sex, BMI, asthma and smoking, CRS remained a risk factor for developing CB. Moreover, for every additional CRS cardinal symptom reported, the risk of having CB increased. An increased number of reported CRS cardinal symptoms could indicate more severe CRS. Subjects in the *CRS+* group reported the most CRS cardinal symptoms and had an odds ratio of developing CB that was seven times higher than subjects not reporting any CRS cardinal symptoms at all. In line with other studies, age, BMI, smoking and asthma were also related to CB [12, 23, 24]. In a small Danish clinical study from 2014, Hakansson et al. [18] reported that CB is more common in CRS patients with nasal polyps compared with controls (42.5% vs. 9.5%, *p* < 0.01). In 2000, Montnémery et al. [19] found an association between CB and upper airway symptoms in a Swedish cross-sectional, population-based study. However, they used a different definition of CB and did not follow the EPOS definition of CRS, since it was not established at the time [2]. To the best of our knowledge, this is the first prospective study to assess upper respiratory symptoms and self-reported CRS based on the EPOS definition and the risk of developing CB in a large, random population sample.

CRS and CB share several risk factors, such as tobacco smoking and occupational exposure [15, 17, 25–27]. The main risk factor for developing CB is tobacco smoking and the cumulative 30-year incidence of CB in current smokers has been reported at 42%, compared with 12% in never-smokers [8], which is in line with our findings. Furthermore, both CRS and CB are associated with asthma [3, 28]. In this study, we adjusted the analyses for asthma and smoking and the relationship remained the same. One hypothesis that could explain the association between CRS and CB is that patients with CRS develop a compromised “gatekeeper” function in the upper airway and less protection of the lower airways because of the ongoing sino-nasal inflammation. This impaired gatekeeper function could expose the lower airways to an excess of unconditioned inhaled air and make the lower airways more susceptible to developing chronic bronchitis. Another possible hypothesis is a systemic spread of inflammation via the bone marrow to both the upper and lower airways through the blood stream, as has been suggested in the well-established relationship between allergic rhinitis and asthma [29, 30]. There is also evidence of an association between CRS and COPD [5, 6, 31, 32]. Even though CB is often associated with COPD, CB is a primary disease entity that often co-exists with COPD and is also considered a phenotype of COPD. This study highlights the importance of conducting further clinical studies investigating a causal relationship between ongoing upper and lower airway inflammation in subjects with CRS and CB.

The Telemark Study is a large, prospective, population-based study of an unselected population. Another strength is the prospective study design, which makes it possible to study the development of CB. Furthermore, the study population is representative, with a prevalence of asthma close to the EU estimate at 8.2% in adults. In the study population, 20% reported to be current smokers which is slightly higher compared to 16% of the general population in Norway in 2013, but that estimate is from all citizens aged 16–74 (Statistics, Norway). In this study, the response rate was 33%. The potential causes and effects of non-response in the Telemark Study were investigated in 2016 by Abrahamsen et al. [20]. They concluded that the estimates for asthma and several respiratory symptoms including a productive cough were valid.

The fact that this study is based on questionnaire data made it possible to address a large population-based cohort. The CRS definition used here is based on the EPOS recommendations for epidemiological, questionnaire-based studies. However, in the absence of a clinical examination of the nose and/or CT scan of the sinuses, it is not possible to exclude the possibility that the CRS cardinal symptoms have origins other than nasal mucosal inflammation, such as structural obstruction of the nose (nasal septal deviation, turbinate hypertrophy) or facial pressure or pain (migraine) etc. In questionnaire-based studies, there is also a risk of recall bias. This questionnaire-based study did not include data on all shown associations with CB and CRS. For instance, CRS is related to allergic rhinitis and CRS with nasal polyps has been associated with genetic factors [33]. It is also important to acknowledge that both CRS and CB can develop and resolve over time. Both CRS and CB are chronic respiratory diseases with a substantial impact on daily life and health-related quality of life for many patients. It is important that clinicians are aware of the close association between upper and lower airway inflammation beyond asthma and allergic rhinitis and more studies are needed to examine possible mechanisms and improved treatments.

## Conclusion

To our knowledge, this is the first large, prospective, population-based study reporting an increased odds of developing CB in subjects with CRS during a five-year observation period. Physicians should be aware of chronic bronchitis in patients with CRS.

## Data Availability

The datasets generated and/or analysed during the current study are not publicly available because of privacy policy regulations, but they are available from the corresponding author in response to a reasonable request.

## Abbreviations

BMI: Body Mass Index
CRS: Chronic rhinosinusitis
CB: Chronic bronchitis
COPD: Chronic obstructive pulmonary disease
EPOS: European position paper on rhinosinusitis and nasal polyposis
